# Investigation of the Tribological Characteristics of Aluminum 6061-Reinforced Titanium Carbide Metal Matrix Composites

**DOI:** 10.3390/nano11113039

**Published:** 2021-11-12

**Authors:** G. B. Veeresh Kumar, R. Pramod, R. Hari Kiran Reddy, P. Ramu, B. Kunaal Kumar, Pagidi Madhukar, Murthy Chavali, Faruq Mohammad, Sachin K. Khiste

**Affiliations:** 1 Department of Mechanical Engineering, National Institute of Technology—Andhra Pradesh, Amrita Vishwa Vidyapeetham University, Coimbatore 641112, India; pmadhu88@gmail.com; 2 Department of Mechanical Engineering, Amrita School of Engineering, Bengaluru Campus, Amrita Vishwa Vidyapeetham University, Coimbatore 641112, India; r_pramod@blr.amrita.edu (R.P.); ChavaliM@gmail.com (R.H.K.R.); farooqm1983@gmail.com (P.R.); kunalkumarb@gmail.com (B.K.K.); 3 Office of the Dean (Research) & Division of Chemistry, Department of Science, Faculty of Science & Technology, Alliance College of Engineering and Design, Alliance University (Central Campus), Chikkahagade Cross, Chandapura-Anekal Main Road, Bengaluru 562106, India; 4 Department of Materials, NTRC-MCETRC, Guntur 522201, India; 5 Department of Chemistry, College of Science, King Saud University, P.O. Box 2455, Riyadh 11451, Saudi Arabia; fmohammad@ksu.edu.sa; 6 Department of Medicine, Harvard Medical School, Boston, MA 02115, USA; skhiste@bwh.harvard.edu

**Keywords:** aluminum, metal matrix composites, titanium carbide, density, strength, wear

## Abstract

The current trend in the materials engineering sector is to develop newer materials that can replace the existing materials in various engineering sectors in order to be more and more efficient. Therefore, the present research work is aimed at fabricating and determining the physical, mechanical, and dry sliding wear properties of titanium carbide (TiC)-reinforced aluminum alloy (Al6061) metal matrix composites (MMCs). For the study, the Al6061-TiC microparticle-reinforced composites were fabricated via the liquid metallurgy route through the stir casting method, where the reinforcement of the TiC particles into the Al6061 alloy matrix was added in the range of 0 to 8.0 wt.%, i.e., in the steps of 2.0 wt.%. The synthesis procedure followed the investigation of the various mechanical properties of Al6061-TiC MMCs, such as the density and structure, as well as mechanical and dry wear experimentation. The tests performed on the casted Al6061, as well as its TiC composites, were in harmony with ASTM standards. As per the experimental outcome, it can be confirmed that the increase in the weight percentage of TiC into the Al6061 alloy substantially increases the density, hardness, and tensile strength, at the expense of the percentage of elongation. In addition, the dry wear experiments, performed on a pin-on-disc tribometer, showed that the Al6061-TiC MMCs have superior wear-resistance properties, as compared to those of pure Al6061 alloy. Furthermore, optical micrograph (OM), powdered X-ray diffraction (XRD), energy dispersive spectroscopy (EDS), and scanning electron microscopy (SEM) analyses were employed for the developed Al6061-TiC MMCs before and after the fracture and wear test studies. From the overall analysis of the results, it can be observed that the Al6061-TiC composite material with higher TiC reinforcement displays superior mechanical characteristics.

## 1. Introduction

In the cutting-edge field of material science and technology research, there are always efforts made for the search and development of newer materials, which has always been considered to be the biggest challenge. Hybrid materials with efficient properties, such as those with superior strength-to-weight ratios, that are lightweight, with good stiffness and flexibility, and that have resistance to cracking and corrosion, have caused researchers to use composite materials as a replacement for conventional materials other than alloys. The performance of composite materials was previously compared with that of normal conventional materials [1,2,3]. As composite materials filled with particulate reinforcement have shown resistance indentation and are largely used in automobile applications, such as cylinder blocks, vibrators, impellers, pistons, calipers, microwave channels, etc., [4], aluminum (Al) alloys are more extensively used in the marine, aerospace, and mineral industries because of their superior mechanical and thermal properties [5]. Among the several Al alloy series, Al6061 is widely used because it is lightweight, formable, and corrosion-resistant, with good electrical and thermal conductivity. Al6061 has found applications in highway fields and the construction industries [6]. In some applications, where the temperature involved is high, the Al6061 can become soft and lose its properties. The hardness of the material has a significant impact on the wear behavior of Al6061 aluminum oxide (Al_2_O_3_) composite systems [7]. Among the various metal matrix composites (MMCs), Al, as the base matrix, improved the mechanical and sliding wear properties [8,9]. It has been proven that the reinforcement percentage, applied load, and sliding distance (SD) have a significant impact on the wear characteristics of MMCs [10,11]. The most valid reason for the failure of various machine components is ‘wear’. A study of wear characterization on Al6061-Al_2_O_3_ composites showed remarkable wear resistance in dry sliding wear processes [12]. It has been clearly observed that the temperature has a significant effect on the wear rate (WR) of composite Al6061-silicon carbide (SiC), and it has been shown that this particular property is reduced with an increase in the applied load [13]. In addition, the same temperature influence has been reported on Al-based composites and wear phenomena [14]. Moreover, the wear behavior of Al6061-Saffil composites was reported, along with an increase in the resistance to wear with an increase in the Saffil content in the base alloy [15]. It was determined that a 15 vol.% of SiC, which was manufactured via powder metallurgy techniques, showed increased wear resistance [16]. When employing TiC particles as reinforcements, it was observed that, during the crystallization of MMCs, Al_3_Ti needles formed, and the TiC particulates served as nucleates, causing the matrix to improve the strength and elastic modulus significantly [17]. A few researchers have also established the reaction between Al, TiC, and Al_4_C_3_ at an equilibrium of 100 °C. These reactions indicate higher TiC stability in the aluminum matrix [18]. TiC appears to have decent wettability and controlled reactivity, as per several research investigations [19]. Because magnesium in Al6061 has little inclination to produce carbides, it did not participate in reaction-aided wetting. However, the production of Mg_2_Si, in addition to CuAl_2_, was detected [20]. As a result, the wettability and reactivity had an impact on the reinforcement/matrix adhesion, and the ultimate properties of the composite were heavily impacted by these characteristics, owing to the transfer of the load from the matrix to the reinforcements [21]. As the TiC weight content increased, the MMCs witnessed an increase in strength and hardness. The specific wear rate of the TiC-reinforced composites was observed to decrease linearly with an increase in the weight percentage of the reinforcements. With a higher normal load and TiC volume fraction, the average coefficient of friction falls linearly [22].

TiC has a higher hardness and elastic modulus, low density, excellent wettability with molten Al6061, and minimal chemical reactivity. As a consequence of their outstanding wear resistance, high strength-to-weight ratios, and superior mechanical characteristics, Al-TiC composites have a distinctive place in the family of ceramic-reinforced MMCs. The quantity and uniform dispersion of the TiC particles in Al-TiC composites are dependent on the development of such characteristics [23,24]. Therefore, keeping in view the importance of fabricating Al-based composites with efficient mechanical properties, the current work deals with the fabrication of Al6061-TiC composites. In this study, we analyzed the effects of different compositions of TiC reinforcements on the density, microstructure, mechanical (tensile strength, hardness, and percentage elongation), and wear properties of an Al6061 matrix alloy.

## 2. Materials and Methods

### 2.1. Materials

For the study, the matrix alloy selected was an Al-Mg-Si series alloy (Al6061 ingots) that was procured from Fenfee Metallurgical Industries (Bangalore, Karnataka, India). The following, Table 1, summarizes the chemical composition of the used Al6061 alloy.

Similarly, TiC, having the particle reinforcement of a 50 µm size, was supplied by Triveni Chemicals (Vapi, Gujarat, India), and the physicochemical properties of the Al6061 matrix and the TiC particles used for the reinforcement are provided in Table 2.

### 2.2. Fabrication of Al6061-TiC MMCs

The fabrication of MMCs through the liquid metallurgy (L/M) route of stir casting is easy and economical, and so we used this stir casting method in the present study for the fabrication of Al6061–TiC composites. The Al6061 alloy was added inside the electrical furnace, which was subjected to heating at a temperature of 710 °C. After reaching the required temperature, the alloy was converted into a molten state, where the Mg chips (1% of the total weight) and the degassing tablets of hexachloroethane (C_2_Cl_6_), of 25–50 g, were added to the molten or liquid. The addition of these agents enhances the wettability and removes gases from the reaction mixture, in addition to allowing the formation of a very thin layer between the molten material and the atmosphere. To ensure the proper mixing of contents, the stirrer was coated with Gr, which has the cup-shaped vanes to stir molten alloy at a speed of 400 rpm. The TiC particles were enfolded into the Al foils, as well as preheated in a muffle furnace up to 350 °C, before being added into the vortex that is formed as a result of stirring (at 400 rpm). Here, a 10-min duration of stirring was followed to make sure that the TiC reinforcement was distributed uniformly in the molten liquid and, after the period, the contents were poured into the preheated cast iron mold boxes (150 mm length and 25 mm diameter). The amount of TiC reinforcement was increased from 0 to 8.0 wt.%, at a rate of a 2% increase each time [25,26].

### 2.3. Instrumental Analysis

The fabricated castings of the Al6061-TiC MMCs were machined using a computerized lathe machine to the required sizes, as per the ASTM standards, in order to study the characteristics, such as the microstructure, density, hardness, % of elongation, strength, and wear resistance. The densities of the alloys and the composites were found using the rule of mixtures (ROM), and the values were compared with the measured weight-to-volume ratios. An optical metallurgical type of microscope model from NIKON, the 150 ECLIPSE, of Japanese make, was used to study the microstructures of the alloy and the MMC specimens. For the powdered X-ray diffraction (XRD) analysis, in the 2θ range of 20–80°, the X’Pert Powder XRD instrument (PANalytical, Almelo, The Netherlands), containing the X’Celerator ultrafast multistrip detector, was used. A scanning electron microscope (SEM), equipped with an energy-dispersive spectroscopy (EDS) detector, JEOL JSM-7100F (at Jain University’s Centre for Nano and Material Science, Bangalore, India), was used to analyze the micropictures of Al6061 and its TiC-filled composites. The resistance to indentation, the “hardness test”, was conducted, utilizing the MRB 250 Brinell hardness tester. The percentage of elongation (ductility), as well as the strength tests of Al6061 and its TiC MMC specimens, were investigated using a computerized tensometer apparatus. The alloy base and the MMC specimens, adapted for tensile strength trials, were machined to get the size specifications, as per ASTM E8 M15a standards. Ducom Instruments (Bangalore, India) make a computerized “pin-on-disc” (POD) tribometer apparatus, which was used to study the wear nature of alloy Al6061 and the TiC-filled composites, with an application of 10 to 60 N loads, in the steps of a 10 N increase. The Al6061, and its TiC MMCs of the cylindrical test specimens used, had the dimensions of 10 mm in length, and 10 mm in diameter. These test samples were prepared to slide under dry conditions against an EN31 steel circular counter face, having a hardness of 60 HRC. For the alloy and the MMC cylinder-shaped samples (10 mm × 10 mm in diameter and height, respectively), an EN31 steel counter disc was adopted. The height loss due to the wear was recorded at a 30 s interval during individual tests, using an accuracy of 1.0 µm on the LVDT (linear variable differential transformer) to plot the graph among the wear height loss and the sliding distance. The counter disc was rotated at a speed of 500 rpm and maintained the SD of 6.0 km. The SEM microphotographs of the alloy and the MMC specimens, subjected to pin-on-disc tests, worn-out surfaces, and tension-test-fractured surfaces, were engaged to analyze the type of fracture that occurred, and the type of wear, as investigated by [27].

## 3. Results and Discussion

### 3.1. EDS, SEM, and XRD Studies of Al6061 and Al6061-TiC MMCs

**Figure 1:**
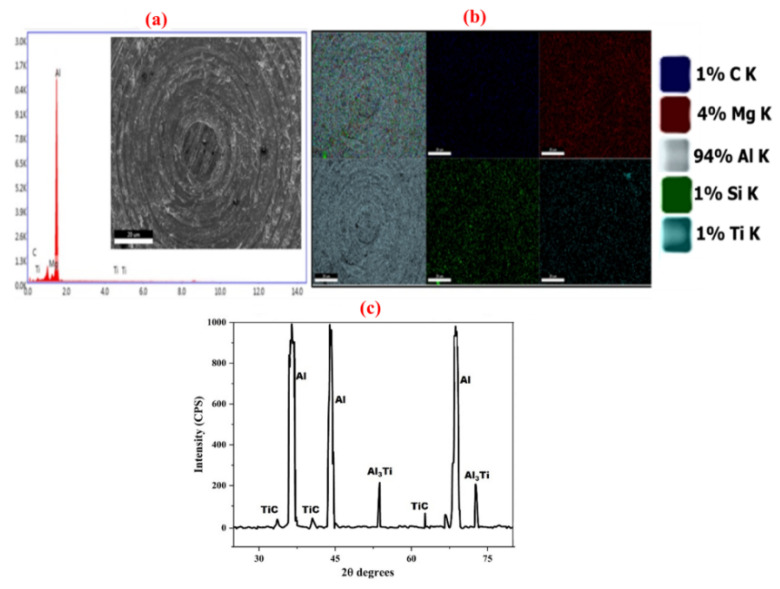
(**a**) SEM image, along with EDS analysis; (**b**) the corresponding elemental mapping using the RUSK model of Al6061-TiC MMC sample; and (**c**) XRD analysis results of Al6061-TIC MMCs.

The matrix alloy used for the current studies was Al6061 reinforced with TiC and was subjected to EDS and SEM investigations to verify the composition of the alloy used; the outcome is provided in Figure 1. As shown in Figure 1a, the observation of elemental peaks for Mg and Si in the pure Al alloy sample, as provided by the EDS analysis, confirms the availability of the respective composition in the sample.

Moreover, the SEM image of the Al6061-TiC MMCs, provided in Figure 1a, was subjected to elemental mapping studies of the RUSK model, using SEM, and clearly shows the availability of Al, along with other alloying elements, Mg and Si, in the matrix of pure alloy, further confirming the presence of other elements into the matrix of the Al6061 alloy. In addition, the mapping of the diffraction peaks for the elements of Al, TiC, and Al_3_Ti in the fabricated Al6061 alloy, provided in Figure 1c, clearly confirms the existence of the respective compounds. Furthermore, from the cumulative analysis of the results, provided in Figure 1b,c, supportive information can be gained for the availability of alloying elements, such as Si and Mg, along with the reinforcing elements, Ti and C, and the base, Al. Finally, the EDS and XRD analyses confirmed the successful fabrication of the Al6061-filled TiC MMCs through liquid metallurgy, via the stir casting method.

### 3.2. Density and Porosity of Al6061-TiC MMCs

**Figure 2:**
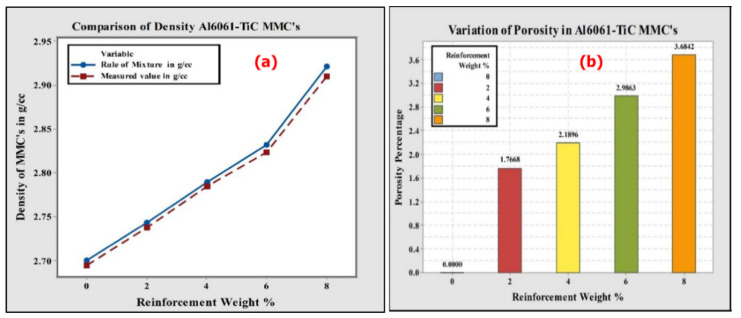
(**a**) Weight-to-volume ratios and thermotical densities; and (**b**) porosity of Al6061-MMCs samples.

The theoretical density values were calculated using the rule of mixtures for different compositions of Al6061-TiC MMCs, and were compared with the practically obtained density values, using weight-to-volume ratios. Both the practical and theoretically obtained densities studied are graphically represented in Figure 2a. From the figure, it is understood that the theoretically obtained density values are higher than the practical density values. This result of the MMCs is explained by the presence of a small amount of casting defects. Nevertheless, from both readings of the densities, it can be observed that the values increased with an increasing percentage of TiC reinforcement in the Al6061 alloy. The increase in density values is due to the blending of the higher-density TiC particles. As can be seen from the figure, this further confirms that the densities of the MMCs are greater than that of the base alloy, similar to those reported in [28].

The percentage porosity of the Al6061 alloy and the TiC-filled MMCs were studied using the Archimedes principle, with the help of Equation (1) mentioned below, and Figure 2b, which presents the variations in the percentage porosity with the increase in the TiC particulate content in the matrix. It can also be noticed from the figure that the percentage porosity values increase with the increase in the TiC particulate content in the Al6061 matrix.

(1) $$\mathrm{Porosity}\;\mathrm{percentage}=\frac{\mathrm{Theoretical}\;\mathrm{density}-\mathrm{Measured}\;\mathrm{density}}{\mathrm{Theoretical}\;\mathrm{density}}\times 100\%$$

The variations in the porosity values of the MMCs can be attributed to the entrapment of gases during the initial mixing, shrinkage at solidification, hydrogen gas evolution, as well as to the entry of air bubbles into the molten composite, or an air envelope around the filled reinforcement particulates of TiC. Thus, the porosity increases with an increase in the weight percentage of the TiC reinforcement, as reported in [29,30].

### 3.3. Microstructure Studies of Al6061-MMCs

**Figure 3:**
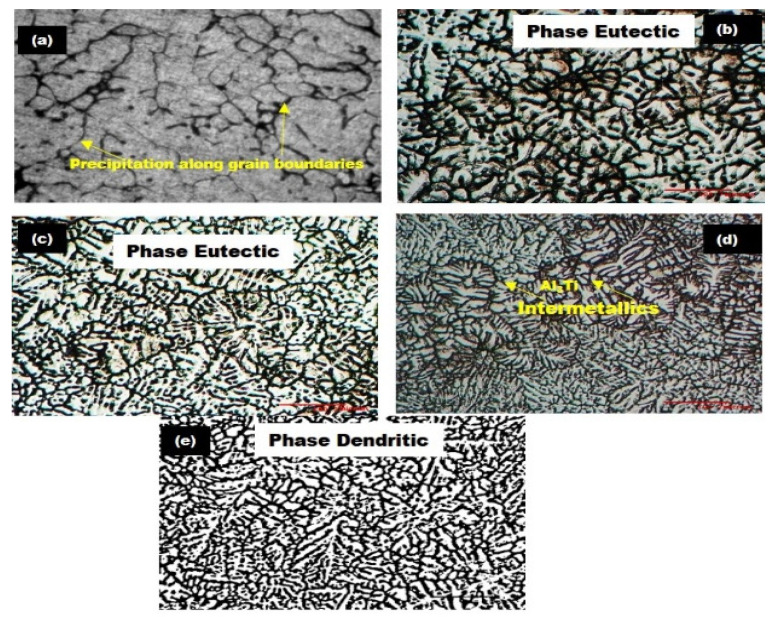
Optical micrographs of Al6061-TiC MMCs at 100× magnification (scale: 100 µm): (**a**) Al6061 base alloy; (**b**) Al6061-2 wt.% TiC; (**c**) Al6061-4 wt.% TiC; (**d**) Al6061-6 wt.% TiC; and (**e**) Al6061-8 wt.% TiC.

Figure 3a–e present the SEM-provided optical micrographs (OM) of the Al6061 base alloy and the Al6061-TiC-filled MMCs, as well as the formation of a more uniform distribution of the TiC reinforcement into the Al6061 alloy, as can be seen from the microphotographs. The microphotographs clearly show an increased TiC filler content in the Al6061 alloy when we move from Figure 3a towards Figure 3e, in addition to the maintenance of a small amount of porosity. The lower porosity of the MMCs can be linked to their higher hardness, as investigated in [31]. Additionally, from the presented microphotographs, it can be seen that there is decent bonding between the Al matrix and the filler TiC particles, therefore resulting in the enhanced transfer of the load from the Al6061 alloy to the filler particles. Furthermore, the microstructure of the Al alloy base, shown in Figure 3a, indicates the presence of a small number of solid solution dendrites of Al, while in Figure 3b–e, the OMs show the same solid solution Al dendrites, with some randomly distributed TiC filler elements, as reported in [32]. This filler content can be visibly shown by increasing the TiC concentration from 0% to 8 wt.%, thereby confirming the success of the synthesis procedure.

**Figure 4:**
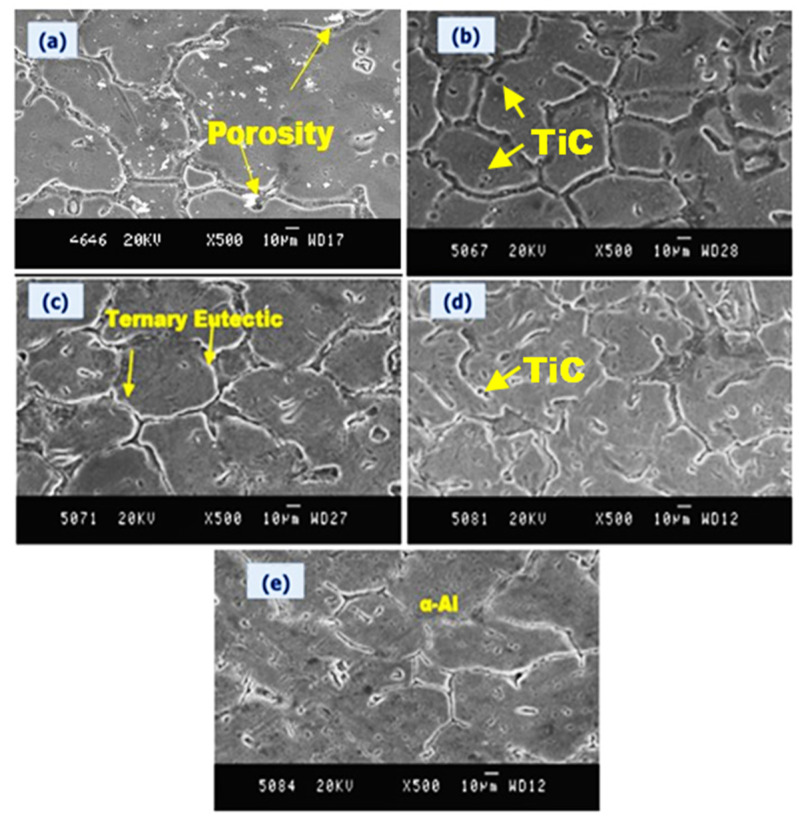
SEM images of Al6061-TiC MMCs at 500× magnification (scale: 10 µm): (**a**) Al6061 base alloy; (**b**) Al6061-2 wt.% TiC; (**c**) Al6061-4 wt.% TiC; (**d**) Al6061-6 wt.% TiC; and (**e**) Al6061-8 wt.% TiC.

Similarly, Figure 4a–e represents the SEM images of the pure alloy and the Al6061-TiC MMCs at different magnifications, with a scale of 10 µm. The OM of the Al6061-TiC MMCs confirms the reinforcement, and even the spread, of the TiC particles in the matrix of Al6061. Moreover, as indicated in Figure 3, there is visual proof of the decent bonding between the the TiC reinforcing phases with the Al6061 matrix. Furthermore, with good interfacial bonding, the reinforcing phase with the matrix phase leads to effective load transfer, thereby supporting the enhanced strength of the MMCs.

### 3.4. Hardness, Tensile Strength, and Ductility of Al6061-TiC MMCs

**Figure 5:**
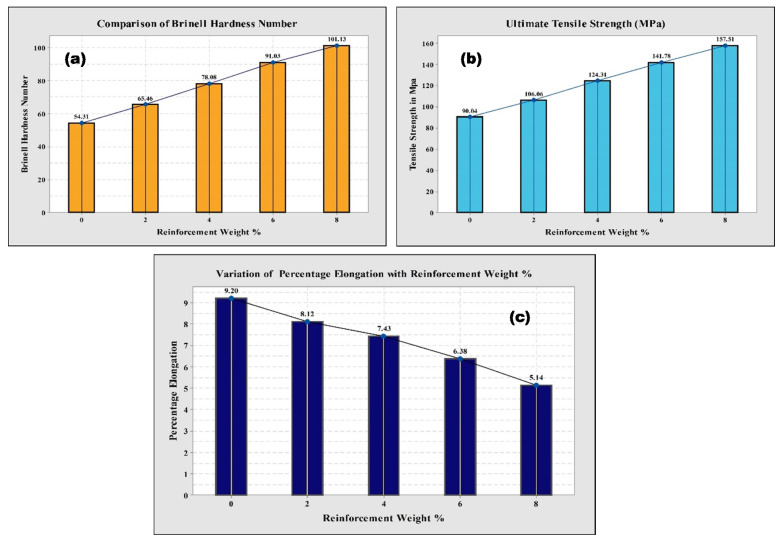
(**a**) hardness of Al6061-TiC MMCs, with increasing wt.% of reinforcement; (**b**) UTS of Al6061-TiC MMCs with wt.% of reinforcement increase; and (**c**) percentage elongation of Al6061-TiC MMCs with the increase in reinforcement.

Hardness accounts for one of the most important characteristics of materials that directly affects the properties of alloys and MMCs, such as strength, toughness, fatigue, and resistance to sliding wear. The resistance to plastic deformation–the hardness test for the base alloy and the MMC specimens, was done with a Brinell hardness tester, and was carried out as per the ASTM E10-07a standard, at room temperature (RT). A hardened steel ball intender with a 100 kg load was followed for hardness evaluation. Each value presented here is an average value of five individual experiments. Figure 5a illustrates the results obtained from the hardness experiments carried out on the Al6061 and its TiC composites. It is apparent from the figure that the Al6061-TiC composites showed higher hardness values than the Al6061 cast alloy. Moreover, as the wt.% of the particle reinforcement increases, the hardness value is also observed to be increasing [33]. The upgradation in hardness readings can be explained by the fact that the higher hardness values of the TiC reinforcement, and its presence in the alloy, Al6061, improve the hardness of the composite. A similar study observed that the presence of harder ceramics in the Al6061 matrix enhanced the hardness properties of the composite [34].

The UTS tests of the fabricated specimens were determined using a computerized tensometer, where the MMC standard specimens used for the tension tests were machined to the required dimensions with the help of a computerized lathe. The dimensions of the UTS test specimens were taken following the E8-M15a standards recommended by ASTM, and the tests were conducted at RT. Furthermore, it was determined that the results obtained were in line with the investigations made. The increase in the strength of the specimens with an increased percentage in the particulate TiC reinforcement is described in Figure 5b. The obtained results of the tensile tests indicate that the tensile strength of the tested specimens increased by 74.5%, with an increase in the percentage of reinforcement varying from 0 to 8 wt.%. The enhancement in the tensile strength values can be due to the higher density, the hardness values of the TiC reinforcement, and the increase in the density and hardness of the Al6061-TiC MMCs, as similarly reported in [35].

Figure 5c indicates the decrement in the percentage elongation of the TiC particle content, which increases with the increasing TiC content, in the range of 0–8%. Almost all of the studied property results of the Al6061-TiC composite materials gradually increased with an increase in the particulate TiC content, but at the cost of the ductility decrement. This reduction in ductility is mainly due to the increase in hardness and density values. Moreover, the reduction in the ductility may be attributed to the fact that TiC is similar to ceramic reinforcement, with its brittle nature [36,37]. It is also confirmed, from the obtained results, that the percentage elongation of the MMCs decreased by an amount of 44.2%, as the filler percentage of the TiC particulate reinforcement increased from 0 to 8 wt.%.

**Figure 6:**
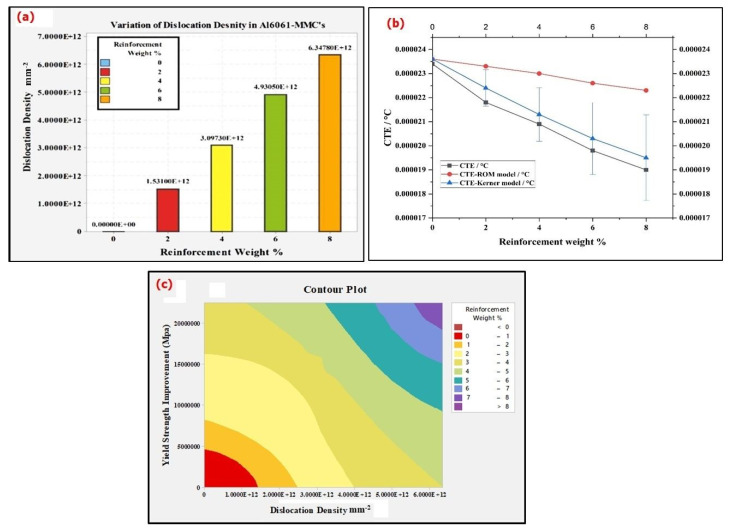
(**a**) dislocation density variation; (**b**) line plot of experimental CTE, Kerner model CTE, and ROM model CTE; and (**c**) variation of yield strength vs. dislocation density of various Al6061-TiC MMC samples.

In the current studies, we confirm that the UTS increase may be explained by the fact that the variation in the dislocation density values was triggered because of the thermal disagreement between the base material and the TiC particulates. Since a large difference can be noticed in the values of the coefficient of thermal expansion (CTE) between the matrix Al6061 alloy (α_Al6061_ = 23.2 × 10^−6^/°C) and the TiC particles (α_TiC_ = 7.7 × 10^−6^/°C), alloy Al6061 affects the thermal stresses for minimal changes in the temperature. This CTE deviation results in the generation of dislocations near the matrix and particle interfaces. Equation (2) denotes the dislocation density [38], where b indicates the Burgers vector (0.32 nm), $V_{r}$ is the volume fraction of the TiC reinforcement, B refers to the geometric constant and is 12 for particulates, ε refers to the strain due to matrix Al6061 and the TiC particle reinforcement thermal mismatch, and d refers to the average grain diameter of the TiC particles (0.55 μm). The dislocation density variation is shown in Figure 6a.

(2) $$\rho =\frac{{B\varepsilon V}_{r}}{\mathrm{bd}\left(1-V_{r}\right)}$$

Furthermore, it can also be seen that the dislocation density increases with a decrease in the CTE. The CTE was determined using the rule of mixtures and the obtained results are represented in Table 3. The dislocations present with a decrease in the CTE, and an increase in the thermal mismatch, helps in the yield strength increase in the MMCs. The dilatometry was performed using TMA-thermomechanical research equipment, from ambient temperature to 450 °C. The thermal expansion of the specimens was calculated by LVDT in multiple consecutive thermal cycles during the expansion, under heating and cooling, at levels of 2 °C/min, under an inert atmosphere. The temperature of the specimen was calculated using a thermocouple, located within the vicinity of the specimen.

Figure 6b presents a line plot of the experimental CTE, using the Kerner model CTE, and the ROM model CTE, as shown in Equations (3) and (4), respectively, for Al6061-TiC MMCs.

(3) $$\alpha_{c}=\alpha_{m}V_{m}+\alpha_{r}V_{p}+V_{m}V_{r}\left(\alpha_{p}-\alpha_{m}\right)\frac{K_{p}-K_{m}}{V_{m}K_{m}+V_{p}K_{p}+3K_{m}K_{p}/4G_{m}}$$

(4) $$\alpha_{C}=\alpha_{m}V_{m}+\alpha_{p}V_{p}$$

Theoretically, the overall CTE value of certain composite materials can be measured by the rule of mixtures, but there are always some errors between the results of the rule of mixtures and the experimental test, as shown in Equation (5):

(5) $$\propto_{C}=V_{p}\left(\mathrm{CTE}\;\mathrm{of}\;\mathrm{Reinforcement}\right)+V_{m}\left(\mathrm{CTE}\;\mathrm{of}\;\mathrm{Matrix}\right)$$

where $\propto_{C}$ is the overall CTE of the composite, and $V_{p}$ and $V_{m}$ are the volume fractions of the reinforcement and the matrix, respectively.

However, the interaction between the reinforcement, the matrix, and the formation of a third intercompound layer between them, whether the composite is in situ or ex situ, and the percentage of pores in the composite drift, result from their theoretical calculations.

Turner Equation (6):

(6) $$\propto_{C}=\left(\propto_{m}K_{m}V_{m}+\propto_{p}K_{p}V_{p}\right)/\left(K_{m}V_{m}+K_{p}V_{p}\right)$$

where $\propto_{C,}$ $\propto_{m}$, and $\propto_{p}$ are the CTEs of the composite, matrix, and the particulates, respectively. $K_{m}$ and $K_{p}$ are the bulk modules of the matrix and the particulates, respectively.

The strengthening results are due to an increase in the dislocation density generated by the difference in the CTEs, and can be evaluated by Equation (7):

(7) $$\sigma =\mathrm{kGb}\left(\rho^{\left(1/2\right)}\right)$$

where σ refers to the yield strength enhancement due to an increase in the dislocation density; k is a constant with a varying value between 0.5 and 1.0; G refers to the shear modulus; and b refers to the Berger vector. The enhancement in the yield strength is shown in Figure 6c.

It is evident that with an increase in the TiC reinforcement, there is an increase in the dislocation density, which contributes to the increase in the yield strength. In the particle-reinforced MMCs, we witness a high variance in the mechanical properties of the dispersed particles and the metal matrix. Because of this, the incoherence is set up, which results in the surge of the dislocation density at the interface of the matrix and reinforced particles. The increase in the dislocation density and the incoherence results in the acceleration of precipitation reactions, and they act as precipitation sites of heterogeneous nucleation [39,40].

**Figure 7:**
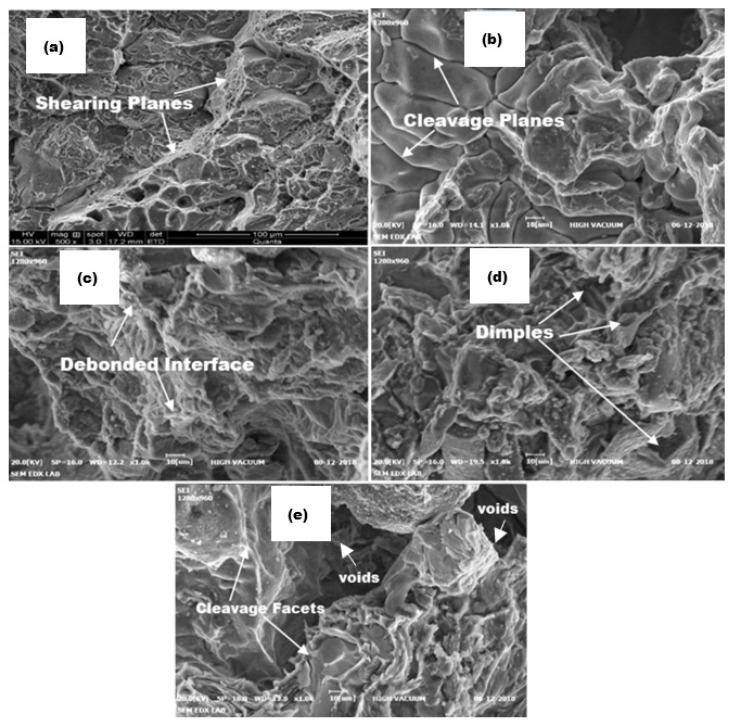
SEM images of the tensile-fractured surfaces of Al6061-TiC MMCs: (**a**) Al6061 base alloy; (**b**) Al6061-2 wt.% TiC; (**c**) Al6061-4 wt.% TiC; (**d**) Al6061-6 wt.% TiC; and (**e**) Al6061-8 wt.% TiC MMCs.

Figure 7 presents the SEM images of the tensile fracture surface of the Al6061-TiC MMCs, where the images show the type of fracture (in Figure 7a), which is more ductile, and the remaining Figure 7b–e are a mixture of ductile and brittle fractures. The loss of ductility with an increase in the hardness and strength characteristics was most noticed in the ceramic-reinforced Al-based MMCs. These results were comparable to those obtained previously.

### 3.5. Wear Studies (Dry Sliding) of Al6061-TiC MMCs

**Figure 8:**
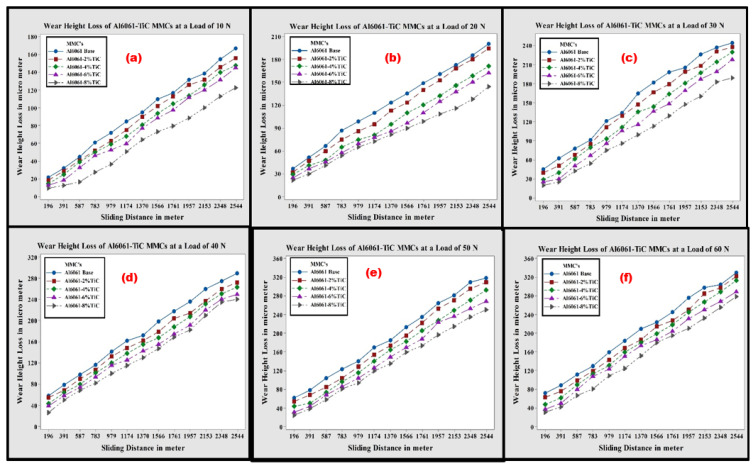
Wear height loss of Al6061 alloy and TiC MMCs, with a 2.5 km SD, at the loads of (**a**) 10 N; (**b**) 20 N; (**c**) 30 N; (**d**) 40 N; (**e**) 50 N; and (**f**) 60 N.

The dry sliding wear resistance of Al6061-TiC MMCs was investigated using a POD tribometer apparatus. Numerous experiments need to be performed on the Al-MMCs in order to determine the dry wear characteristics of the composites, and several researchers have submitted reports related to dry sliding wear in the past couple of decades. In the current study, dry sliding wear tests were performed at RT on the fabricated MMCs. The ASTM standards followed for the dry wear test were according to the standards of ASTM-G99, and maintained the constant sliding velocity. The obtained results from the dry sliding wear tests were adopted to plot various graphs between the wear height losses in microns due to dry wear, and at an SD of 6.0 km on the applied loads from 10 to 60 N, in steps of 10 N. Figure 8a–f represent the dry wear height losses taking place at different SDs, and with different applied loads. From the figures, it is clear that the wear height loss increases with an increase in the SD. Because of the increase in the dry SD, there were changes in the frictional forces, which triggered the Al6061-TiC MMCs’ rise in temperature. The Al6061 alloy and its MMCs become softer as the temperature increases because of the sliding and, therefore, results in a higher amount of wear height loss. In Figure 8a, upon the application of 10 N loads, the height loss reduced gradually because of wear, with an increase in the TiC reinforcement amount in the matrix. This is reminiscent of the enhancement in the hardness of Al6061-TiC MMCs, as discussed earlier.

As presented in Figure 8, at all the sliding distances considered, the dry sliding wear height loss of the Al6061-TiC MMCs was significantly lower compared to the matrix alloy, and it decreased with the increased TiC filler content in the composite material. This can be attributed to the enhanced hardness values of the Al6061-TiC composites. The increase in hardness results in an enhancement of the wear, as well as the seizure resistance of MMCs [41].

**Figure 9:**
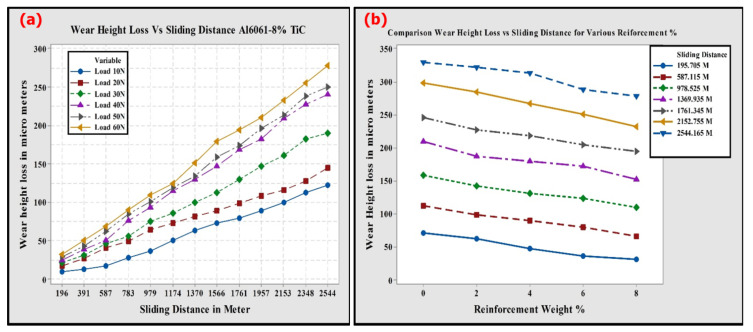
(**a**) the consequence of load on the wear height loss with SD increase on Al6061-8 wt.% of TiC MMCs; (**b**) wear height loss of Al6061 and TiC-filled MMCs with percentage (%) increase of TiC at a load of 60 N.

The variations in the wear height losses with different applied loads are shown in Figure 9a. The applied normal loads largely affected the wear rates of the Al alloys and their MMCs more significantly and was the most significant factor in governing the wear behavior of the alloys and the MMCs [42]. The wear rate of the materials varies linearly and is summarized [43] with the applied load; it is indicative of Archard’s law and significantly lower in-cases of the composite materials [44]. Moreover, with an increase in the normal loads, the base alloy and the composites suffer higher wear height losses. Nevertheless, at all the applied loads, the wear resistance offered by the composites was far superior to the base material. Furthermore, the increased applied loads result in a set of delamination, leading to more severe wear height loss of the monolithic and the composites, as noticed with other composites [45,46].

The variations in the wear height losses of Al6061 and its TiC-filled composites, with an increase in the TiC particulate contents, are presented in Figure 9b. It can be highlighted that the wear height loss of the Al6061-TiC MMCs declines with an increase in the TiC particulate content in the base Al alloy. Nevertheless, for a given filler TiC particulate content, the Al6061-TiC MMCs have lower wear height loss than the base Al6061 alloy. This enhancement in the composite’s wear resistance with increased TiC reinforcement content can be attributed to the improvement in the hardness of the Al6061-TiC MMCs. The improved hardness values affecting the decrease in the wear rate is described in [47,48].

**Figure 10:**
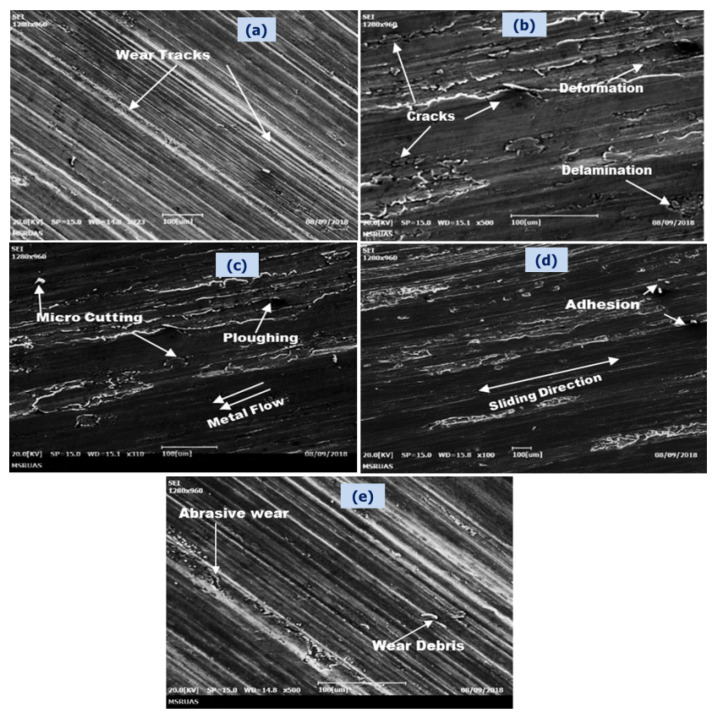
Worn-out SEM photographs of Al6061 alloy and its TiC MMCs after sliding a distance of 6.0 km, with a velocity of 2.62 m/s, at an applied load of 60 N: (**a**) Al6061 alloy; (**b**) Al6061-2 wt.% TiC; (**c**) Al6061-4 wt.% TiC; (**d**) Al6061-6 wt.% TiC; and (**e**) Al6061-8 wt.% TiC.

Figure 10 presents the worn-out surface SEM photographs of cast Al6061 and the particulates of TiC-filled MMCs, at a load of 60 N and a 6.0 km SD. At the 60 N applied load, the composites of Al6061-8 wt.% TiC display lower wear height loss. At higher applied loads, the degree of grooves formed on the worn-out surfaces of the Al6061 base, as well as the MMCs containing lower weight fractions of the TiC particle reinforcement, are relatively larger and undergo severe plastic deformation, resulting in rapid severe wear. This behavior of the materials is evident from the SEM microphotographs, as shown in Figure 10a–e. As can be seen from the figures, the extent of grooving on the Al6061-TiC MMCs’ worn-out surfaces was reduced upon increasing the TiC filler, indicating the lower material loss due to the wear.

The material removal rate and subsurface deformations during the material wear are governed by parameters, such as the microstructure, strength, work hardening rate, hardness, reinforced particle distribution, deformation, and ductility. Because of the uniform distribution of the dispersed reinforced particles of TiC within the Al6061 matrix, the bonding increased the hardness of the MMCs and caused a reduction in the efficiency of the cutting action by the EN31 disc material. At a smaller applied load, the wear is due to the cutting action, and we can observe negligible deformation at the subsurface level. The specimens wear uniformly when subjected to a 30–50 N load. Because of the cavitation decohesion occurring in the sliding direction of the surface, the composite shows lesser permanent deformation and cracks [49]. When the load is medium, the formation of oxide is less than when the load is low, i.e., 10–20 N. We can observe that there is no ploughing of the reinforcement particles of TiC, and they remain adhered to the Al6061 matrix at smaller applied loads. At higher applied loads of 60 N, the deformation at the subsurface level becomes evident, and these deformations are a result of the cutting and ploughing actions [50]. As these subsurface deformations increase, they form microcracks, and some cracks are generated along with the transverse and longitudinal directions. Delamination appears for longer sliding distances than for shorter sliding distances. It can be determined from this that the wear contact interface temperature increases with the increasing sliding distance [51]. The velocity increases the thermal profile of the sliding surface, causing TiC particles to escape the surface layer, and demonstrating that the sliding speed is related to the wear rate, and an abrasive effect is evident, as shown in Figure 10e. As the sliding speed increases, the material degrades fast, owing to the frictional heat generated between the rubbing surfaces [52]. Wear mechanisms differ, as the parameters affecting the wear are the applied load and sliding distance. Adhesion, abrasion, ploughing, and delamination wear are some of the wear processes witnessed in the wear of Al6061-TiC MMC’s. Deformation of the Al6061matrix causes works hardening. As a consequence of this, there is a material removal and cracks appear in the interfacial region of the Al6061 matrix and the TiC reinforcement. Metal-to-metal interactions are prevented by the oxide film. The hard brittle oxide that forms on Al protects it from wear at a 60 N load, and this is evident from the wear tracks observed [53]. These cracks result in delamination wear and they increase with an increase in the applied load. The larger the sliding distances, the more ploughing action is witnessed, in comparison to shorter sliding distances. It may be inferred, therefore, that the interfacial contact temperature of the wear surface increases with an increase in the sliding distance. Smaller loads also lead to wear cracks formed on the worn surface [54].

## 4. Conclusions

In the present study, the stir casting technique (L/M) was successfully utilized in the fabrication of Al6061-TiC MMCs by varying the TiC filler contents up to 8 wt.%. The Al6061-TiC MMC density values were found and noticed to be more improved than their matrix base alloy. The EDS studies conducted on the matrix Al6061 and its TiC particulate-filled MMCs confirmed the presence of the alloying elements in the alloy, and the TiC reinforcement elements in the Al6061-TiC MMCs, confirming the fabrication of MMCs. The OM and SEM studies reported the uniform distribution of the reinforcement TiC particulates in the base material. The hardness and tensile strength properties of the Al6061-TiC MMCs were found to be increased with increased filler content, and Al6061-8 wt.% TiC composites displayed superior hardness and tensile strength values to the remaining MMCs, considered with the decrement in the readings of ductility. The dry wear test results indicate that the Al6061-TiC MMCs offered higher wear resistance than the base alloy. Moreover, the increased loads and the SDs in the wear test reported a higher loss of material and, from the overall investigations and findings, it may be concluded that the Al6061-8 wt.% TiC exhibits significantly improved physical, mechanical, and tribological behaviors in comparison with the other counterparts of Al6061-TiC MMCs.

## Figures and Tables

**Table 1:** Al6061 base material composition.

Chemical Elements	Mn	Si	Cu	Fe	Cr	Mg	Ti	Zn	Al
Alloy Al6061 (wt.%)	0.03	0.62	0.22	0.23	0.22	0.84	0.1	0.10	Bal

**Table 2:** Al6061 and TiC particle reinforcement properties.

Material	Density (g/cc)	Hardness (Rockwell)	Yield Strength (MPa)
Al6061 Alloy	2.7	40	255
TiC (single crystal)	4.92	92	20 × 10^3^

**Table 3:** CTE and dislocation density values of Al601-TiC MMCs.

wt.% of Reinforcement	CTE/°C	CTE-ROM Model /°C	Kerner Model /°C	Dislocation Density mm^−2^
0	0.0000234	0.0000236	0.0000236	0.00000 × 10^+00^
2	0.0000218	0.0000233	0.0000224	1.53100 × 10^+12^
4	0.0000209	0.0000230	0.0000213	3.09730 × 10^+12^
6	0.0000198	0.0000226	0.0000203	4.93050 × 10^+12^
8	0.0000190	0.0000223	0.0000195	6.34780 × 10^+12^

## Data Availability

Can be obtained upon request.
